# Inhibition of angiogenic and non-angiogenic targets by sorafenib in renal cell carcinoma (RCC) in a RCC xenograft model

**DOI:** 10.1038/bjc.2011.55

**Published:** 2011-03-15

**Authors:** J S P Yuen, M Y Sim, H G Siml, T W Chong, W K O Lau, C W S Cheng, H Huynh

**Affiliations:** 1 Department of Urology, Singapore General Hospital, Singapore; 2 Laboratory of Molecular Endocrinology, Division of Molecular and Cellular Research, National Cancer Centre, Singapore

**Keywords:** renal cell carcinoma, sorafenib, molecular targeted therapy, xenograft, survivin

## Abstract

**Background:**

It is widely recognised that sorafenib inhibits a range of molecular targets in renal cell carcinoma (RCC). In this study, we aim to use patient-derived RCC xenografts to delineate the angiogenic and non-angiogenic molecular targets of sorafenib therapy for advanced RCC (aRCC).

**Methods:**

We successfully generated three patient RCC-derived xenografts in severe combined immunodeficient mice, consisting of three different RCC histological subtypes: conventional clear cell, poorly differentiated clear cell RCC with sarcomatoid changes, and papillary RCC. This study also used clear cell RCC cells (786-0/EV) harbouring mutant VHL to investigate the clonogenic survival of cells transfected with survivin sense and antisense oligonucleotides.

**Results:**

All three xenografts retain their original histological characteristics. We reported that sorafenib inhibited all three RCC xenograft lines regardless of histological subtypes in a dose-dependant manner. Sorafenib-induced growth suppression was associated with not only inhibition of angiogenic targets p-PDGFR-*β*, p-VEGFR-2, and their downstream signalling pathways p-Akt and p-ERK, cell cycle, and anti-apoptotic proteins that include cyclin D1, cyclin B1, and survivin but also upregulation of proapoptotic Bim. Survivin knockdown by survivin-specific antisense-oligonucleotides inhibited colony formation and induced cell death in clear cell RCC cells.

**Conclusion:**

This study has shed light on the molecular mechanisms of sorafenib in RCC. Inhibition of non-angiogenic molecules by sorafenib could contribute in part to its anti-tumour activities observed *in vivo*, in addition to its anti-angiogenic effects.

Renal cell carcinoma (RCC) is the most lethal of all urological cancers. Approximately 20–30% of patients with RCC exhibit visceral metastasis at the time of diagnosis (Griffin *et al*, 2007) and of all patients with clinical organ-confined disease, who had undergone potentially curative radical nephrectomy, 30% will develop metastatic disease on follow-up (Zisman *et al*, 2002). Until recently, the only effective treatment for metastatic RCC was cytokine-based immunotherapy with interferon-*α* or interleukin-2, which produce a response rate of only 10–15%. The advent of molecular targeted therapy in the last few years has revolutionised treatment of patients with metastatic RCC with benefits in terms of disease stabilisation, improvement of quality of life, and, in the case of sunitinib, overall survival (Atkins *et al*, 2004; Escudier *et al*, 2007; Heng *et al*, 2009; Motzer *et al*, 2009).

Sorafenib is a small molecule tyrosine kinase inhibitor (TKI) currently FDA approved for advanced RCC (aRCC). It is however not tyrosine kinase receptor (TKR) specific. Indeed, sorafenib (BAY 43-9006) was initially developed as a Raf inhibitor in the class of bis-aryl ureas that inhibits several RTKs, including VEGFR-2 and -3, PDGFR-*β*, FLT3 receptor, and c-KIT receptor (Wilhelm *et al*, 2004; Fabian *et al*, 2005). Although many trials reported clinical efficacies of this new therapeutic, the exact molecular mechanism(s) accounting for its clinical effects is still largely unknown. In addition, there are currently no reliable molecular biomarkers available to predict for treatment responses to this new class of therapeutics. A tumour xenograft model provides a means for pre-treatment drug testing, which allows the study of molecular changes in tumour in response to treatment, which hopefully will lead to better understanding of the molecular mechanisms underlying the clinical activity of sorafenib in RCC.

In this study, we developed RCC-derived xenografts in severe combined immunodeficient (SCID) mice and use them to assess tumour response to sorafenib, and to assess whether treatment responses is RCC subtype dependant. Second, we aim to gain insight into sorafenib-induced molecular changes in the TKR expression profile and the associated downstream signalling pathways.

## Materials and methods

### Drugs and reagents

Research-grade Capsitol was purchased from CyDex, Inc., Lenexa, KS, USA. Primary antibodies against CD31 and Ki-67 were from Lab Vision, Fremont, CA, USA. Anti-cleaved caspase-3, anti-cleaved PARP, Akt, Bim, PDGFR-*β*, and phosphorylation-specific antibodies against eIF4E Ser209, ERK1/2 Thr202/Tyr204, and Akt Ser473 were obtained from Cell Signalling Technology (Beverly, MA, USA). Antibodies against cyclin D1, cyclin B1, cyclin A, cdk-2, cdk-4, cdk-6, p27, Bax, Bad, Bcl-xL, Mcl-1, survivin, ERK1/2, VEGFR-2, phospho-VEGFR-2 Tyr951, phospho-PDGFR-*β* Tyr1021, and *α*-tubulin were from Santa Cruz Biotechnology Inc. (Santa Cruz, CA, USA). Sorafenib tosylate (BAY 43-9006, Nexavar, Bayer and Onyx Pharmaceuticals) was purchased from Bayer HealthCare (Leverkusen, Germany). Sorafenib was dissolved in vehicle (30% Capsitol in water) before use.

### Creation of human RCC-derived xenografts in SCID mouse

Prospectively over 1 year (July 2008–July 2009), patients undergoing nephrectomy for RCC were recruited in the study with informed consent and with approval from our Institution Review Board. In addition, approval was granted by our Institutional Animal Care and Use Committee for *in vivo* experiments involving animals. The conduct of all experiments involving mice adhered strictly to the standard as outlined in the guidelines for the welfare and use of animals in cancer research by Workman *et al* (2010).

Specimens from nephrectomy performed for RCC were obtained intraoperatively. The diagnoses of RCC were confirmed by histology in all cases. Renal cell carcinoma xenografting was carried out with mice (Animal Resource Centre) that are homozygous for the SCID mutation as previously described (Huynh *et al*, 2006). Briefly, freshly sectioned RCC tissues were placed in RPMI 1640 in an ice bath immediately on tumour sectioning. Thin slices of tumour, taken between contiguous thin slices used for cryostat section and permanent sections (paraffin-embedded sections), were weighed, diced into 2–3 mm^3^ pieces, and washed three times with RPMI 1640 medium. They were minced finely to fragments that passed through an 18-gauge needle. They were then mixed 1 : 1 (V/V) with Matrigel (Collaborative Research, Bedford, MA, USA) to give a total volume of 0.2 ml per injection. The tissue mixture was subcutaneously injected in the right flank of 8-week-old male SCID mice. For each tumour, five mice were used. Growth of established tumour xenografts was monitored at least twice weekly for 5 months. For serial transplantation, tumour-bearing animals were killed by CO_2_ exposure. Animals were placed in ice water bath for 2 min. They were then dipped in and out of 10% Clorox (The Clorox Company, Oakland, CA, USA) solution for 2 min, washed in four changes of ice water, placed in 70% ethanol, and transferred to a laminar flow hood for dissection. Tumours were minced under sterile conditions as described above. Fragments that passed through an 18-gauge needle were mixed with Matrigel for serial transplantation in successive SCID mice.

### Testing the efficacy of sorafenib on RCC xenografts

Drug testings were carried out to assess the efficacy of sorafenib in RCC. In brief, mice bearing indicated xenografts (10 per group) were orally administered 200 *μ*l of vehicle (30% Capsitol), or 40 mg kg^−1^ per day sorafenib. Growth of established xenografts was monitored at least twice weekly by Vernier caliper measurement of the length (a) and width (b) of the tumour. Tumour volume was calculated as (a−b^2^)/2. Animals were killed at a pre-determined duration after the last treatment dose, and body and tumour weights were recorded, with tumours collected for further molecular analyses. To study the effects of therapeutic agents on apoptosis cascade activation and TKR signalling pathway activation, mice bearing RCC tumours were treated with vehicle or drugs per kilogram of body weight as described above. Animals were killed at 3 h after the last dose, and tumours were collected and frozen in liquid nitrogen for later analysis to correlate drug responses with tumour biology. Part of the tumour collection was fixed in neutral buffer containing 10% formalin for immunohistochemistry.

For dose–response experiments, 12 clear cell RCC (RCC-07-0408) tumour-bearing SCID mice were randomly divided equally into four groups. Mice bearing tumours were orally treated with 200 *μ*l of vehicle (30% Captisol) or two doses of sorafenib (20 and 40 mg kg^−1^) daily for 21 days, when tumours were approximately 150–180 mm^3^. Tumour growth was determined as described above. Animals were killed on day 21 of treatment and their tumour weight was recorded.

### Antisense survivin treatment

Antisense survivin oligonucleotide (ASO) (kindly provided by Eli-Lilly, Indianapolis, IN, USA) is the sodium salt of a synthetically derived 18-mer phosphorothioate oligonucleotide composed of eight methoxyethyl-modified ribonucleotides and 10 deoxy-ribonucleotides (LY2181308). The sequence of nucleotides is complementary to mRNA involved in the biosynthesis of survivin. LY2181308 hybridises in an antisense orientation to the survivin mRNA (Accession # NM_001168) at position 1099–1116. mismatch control oligonucleotide (MM control LY2293329) for the LY2181308 sodium was prepared by incorporating six mismatch bases while maintaining the same G/C content as that of the antisense molecule.

For gene silencing experiments by survivin ASO, 786-0 cells were transfected with LY2181308 sodium or control LY2293329 using LipofectAmine 2000 in Opti-MEM medium (Invitrogen, Carlsbad, CA, USA). Samples were collected after 72 h and analysed by immunoblotting or flow cytometry analysis.

### Western immunoblot analysis

To determine changes in indicated proteins, eight independent tumours from vehicle- or drug-treated mice were homogenised. Lysates of four tumours from one group were pooled. Each lane represented one protein pool (80 *μ*g of proteins) and two pools per group were subjected to western blotting as described (Huynh *et al*, 2006).

### Immunohistochemistry

Tumour tissue samples were processed for paraffin embedding and 5 *μ*m sections were prepared. The sections were incubated overnight at 4°C with the primary antibodies against CD31, Ki-67, and cleaved PARP as described previously (Huynh *et al*, 2008).

### Clonogenic survival assay

Clonogenic assays were performed as described (Rochester *et al*, 2005). In brief, at 48 h after survivin-specific ASO transfection, cells were re-seeded in 10 cm dishes at 1500 cells per dish. The remaining cells were pelleted by centrifugation at 13 000 r.p.m. at 4°C, washed with ice-cold PBS, and lysed for immunoblotting to quantify survivin gene silencing. Clonogenic assay dishes were incubated at 37°C in 5% CO_2_ for 10–14 days until discreet colonies were visible. Visible colonies were fixed in methanol/acetic acid (3 : 1), stained with crystal violet (400 *μ*g ml^−1^; Sigma, St Louis, MO, USA), and counted on an automated colony counter (ColCount, Oxford Optronix, Oxford, UK).

### Statistical analysis

The graphing and statistical analysis software Prism v.4.0 (GraphPad, La Jolla, CA, USA) and Excel (Microsoft, San Diego, CA, USA) were used to plot and analyse data. Graphs were plotted to show mean values and error bars. Error bars depict standard error of the mean (s.e.m.). The Student's *t*-test and analysis of variance with Bonferroni *post hoc* test were used for the comparison of mean values between two and multiple (>2) data sets, respectively. For the *in vivo* experiments, changes in body weight and tumour weight at killing, mean vessel density, Ki-67 index, and apoptotic cells were compared using Student's *t*-test. A minimum of 95% level of significance (i.e., a *P*-value of less than 0.05) was used to define statistical significance.

## Results

**Figure 1:**
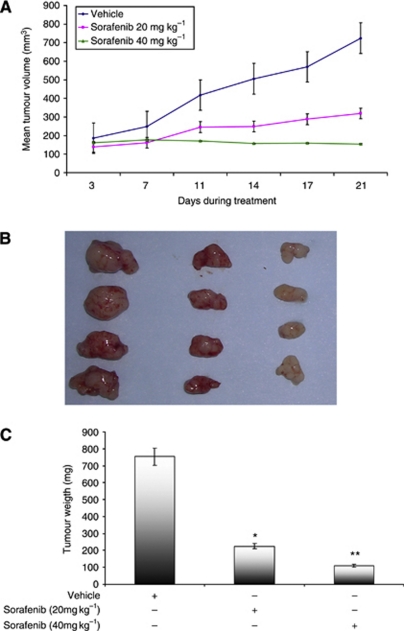
Effects of sorafenib on the growth of RCC-07-0408 xenograft. Mice bearing RCC-07-0408 tumours were randomised (10 mice per group) and treated with vehicle or two doses of sorafenib (20 mg and 40 mg kg^−1^ per day) for 21 days. Mean of tumour volume±s.e. at given time points (**A**), representative vehicle- and sorafenib-treated tumours (**B**), and the corresponding tumour weight (**C**) for RCC-07-0408 xenograft are shown. The asterisks (^*^ and ^**^) indicated significant differences between the vehicle- and sorafenib-treated tumours (*P*<0.05 and *P*<0.001 respectively, analysis of variance). Experiments were repeated twice with similar results.

**Figure 2:**
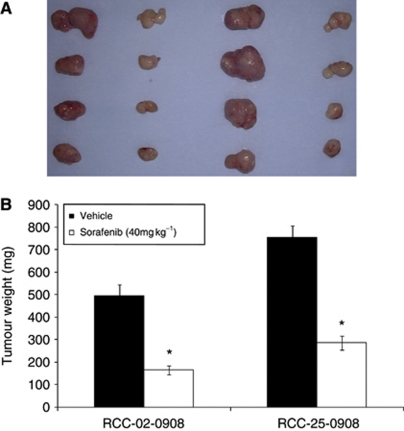
Effects of sorafenib on the growth of RCC-02-0908 and RCC-25-0908 xenografts. Mice bearing indicated tumours were randomised (10 mice per group) and treated with vehicle or 40 mg kg^−1^ sorafenib for 21 days. Representative vehicle- and sorafenib-treated tumours (**A**), and the corresponding tumour weight (**B**) for indicated xenografts are shown. The asterisks (^*^) indicated significant differences between the vehicle- and sorafenib-treated tumours (*P*<0.05, analysis of variance). Experiments were repeated twice with similar results.

Three RCC xenografts derived from nephrectomised RCC specimens were established. Supplementary Figure 1S shows the histological phenotype of xenografts RCC-07-0408, RCC-02-0908, and RCC-25-0908, which were conventional clear cell, papillary, and poorly differentiated clear cell RCC with sarcomatoid changes, respectively. In comparison with the histological features of the clinical specimens, the three established xenografts retain identical histological characteristics compared with the original tumour. We next proceeded to evaluate the ability of sorafenib to suppress the growth of patient-derived RCC xenograft RCC-07-0408, which is of clear cell histological subtype. As illustrated in Figure 1, the growth rate of RCC-07-0408 was inhibited by sorafenib in a dose-dependent manner (Figure 1A). Tumour weights of mice treated with 20 and 40 mg kg^−1^ sorafenib for 21 days were ∼26 and 15% of that treated with vehicle, respectively, (Figures 1B and C, *P*<0.01). Similarly, treatment of RCC-02-0908 (papillary RCC) and RCC-25-0908 (poorly differentiated clear cell sarcomatoid RCC) xenografts with 40 mg kg^−1^ sorafenib also resulted in significant growth inhibition (Figure 2, *P*<0.01). At a dose of 40 mg kg^−1^, sorafenib did not elicit any overt toxicity, as manifested by weight loss, unkempt appearance, mortality, and distress behaviour, in all sorafenib-treated animals during the course of treatment.

**Figure 3:**
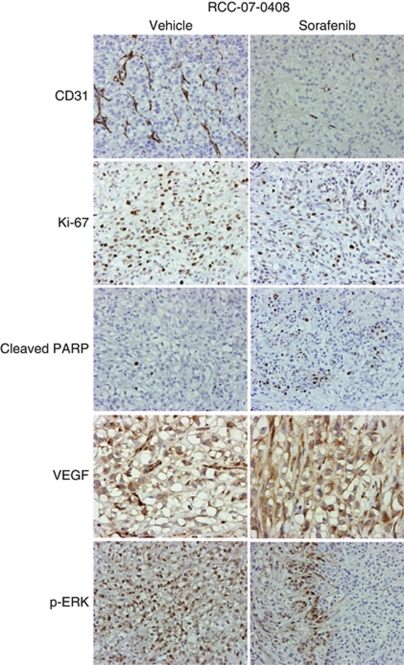
Effects of sorafenib on phospho-ERK1/2, VEGF expression, angiogenesis, cell proliferation and apoptosis of RCC-07-0408 xenograft. Mice bearing RCC-07-0408 tumours were randomised (10 mice per group) and treated with vehicle or 40 mg kg^−1^ per day sorafenib for 21 days. Representative pictures of blood vessels stained with anti-CD31, proliferative cells stained with anti-Ki-67, apoptotic cells stained with anti-cleaved-PARP, VEGF expression stained with anti-VEGF, and p-ERK1/2 stained with anti-phospho-ERK antibodies in vehicle- and drug-treated tumours are shown ( × 200). Experiments were repeated twice with similar results.

We next examined the anti-angiogenic, anti-proliferative, and apoptotic effects of sorafenib in treated tumour xenografts. Representative CD31, Ki-67, cleaved PARP, VEGF, and p-ERK1/2 immunohistochemical stainings for vehicle- and sorafenib-treated RCC-07-0408 and RCC-02-0908 tumour xenografts were shown in Figure 3. Sorafenib-treated xenografts demonstrated significant decrease in mean percentage of CD31-positive endothelial cells in both xenografts compared with vehicle-treated xenografts (*P*<0.05). In relation to this, there was no difference in the expression of VEGF between the sorafenib- and vehicle-treated xenografts, however, the former demonstrated significant decrease in p-ERK staining compared with the latter (*P*<0.05). This finding is consistent with inhibition by sorafenib of the angiogenic pathway mediated by VEGFR and PDGFR in endothelial cells. In addition, there was a significant increase in apoptosis (percentage of cleaved PARP-positive cells) and a significant decrease in proliferation (percentage of Ki-67-positive cells) (*P*<0.05) observed in both sorafenib-treated xenografts compared with the vehicle-treated control. Similar results were obtained when RCC-25-0908 tumours were analysed (Supplementary Figure 2S).

**Figure 4:**
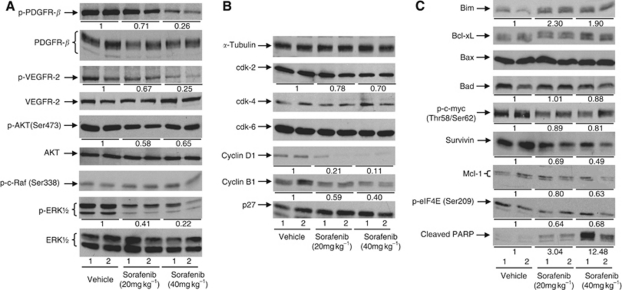
Effects of sorafenib therapy on the phosphorylation of VEGFR-2, FGFR-1, PDGFR-*β*, ERK pathway, Akt, (**A**) expression of cell cycle regulatory proteins (**B**), and apoptosis (**C**) in RCC-07-0408 xenografts. Mice bearing RCC-07-0408 tumours were randomised (10 mice per group) and treated with vehicle or two doses of sorafenib (20 mg and 40 mg kg^−1^ per day) for 21 days. Lysates of four tumours from one group were pooled. Each lane represented one protein pool and two pools per group were subjected to western immunoblot analysis as described under the Materials and Methods section. Representative blots are shown. Experiments were repeated twice with similar results.

To gain insight into the mechanistic actions of sorafenib in RCC, we determined whether sorafenib could effectively inhibit its targeted receptors: VEGFR-2 (Flk1), and PDGFR-*β*. As shown in Figure 4A and Supplementary Figure 3S, sorafenib was clearly effective in inhibiting the phosphorylation of VEGFR-2 Tyr951 and PDGFR-*β* Tyr1021. As sorafenib has been shown to target the Raf/MEK/ERK pathway (Wilhelm *et al*, 2004; Liu *et al*, 2006), we proceeded to examine whether sorafenib-induced tumour growth suppression in RCC-derived xenografts was associated with inactivation of this signalling pathway. Figure 4 shows that although p-c-Raf Ser338 levels were not significantly altered, the levels of p-ERK1/2 and p-AKT Ser473 in sorafenib-treated tumours were lower than those observed in vehicle-treated tumours. These findings are in contrast to our previous observation that sorafenib induces paradoxical ERK signalling pathway in gastric carcinoma through upregulation of p-c-Raf Ser338 (Yang *et al*, 2009). The exact mechanistic explanation for this phenomenon in gastric carcinoma is unclear at the moment. However, our finding showing sorafenib-induced inhibition of both PI3K/AKT and MAPK/ERK pathways in RCC is consistent with sorafenib as a TKI.

As cell cycle proteins might have a significant role in the development of RCC, we evaluated the effect of sorafenib in regulating cell cycle proteins (Figure 4B and Supplementary Figure 3S). Our data showed that although expression of cdk-4, cdk-6, and p27 was not affected by sorafenib, the levels of cyclin D1, cyclin cdk-2, and cyclin B1 in sorafenib-treated tumours were significantly reduced as compared with controls (*P*<0.05). These findings suggest that inhibition of cell cycle progression *in vivo* may prove to be one of the mechanisms accounting for the clinical effects of sorafenib in RCC. We next proceeded to investigate the effect of sorafenib on apoptosis induction. Figure 4C and Supplementary Figure 3S demonstrated that sorafenib also induced profound apoptosis as evidenced by the generation of PARP cleavage products. Although the proapoptotic protein Bim was significantly increased in sorafenib-treated xenograft, expression of the anti-apoptotic protein Mcl-1 was reduced (Figure 4C; *P*<0.05). There are no significant alterations in the levels of Bax, Bad, p-c-myc Thr58/Ser62, p-eIFG4E Ser209, and Bcl-xL following sorafenib treatment. In addition, we found significant downregulation of survivin in tumours treated with sorafenib compared with controls (Figure 4C and Supplementary Figure 3S). The magnitude of change of survivin parallels tumour growth inhibition.

**Figure 5:**
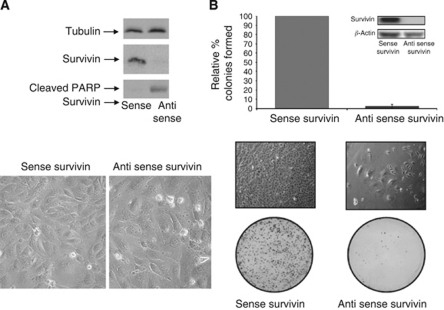
Effects of survivin knockdown on anchorage-dependency, cytokinesis, expression of cell cycle regulatory proteins, and apoptosis in 786-0 cells. 786-0 cells were transfected with sense and antisense survivin as described under the Materials and methods section. At 48 h after transfection, cells were collected, extracted, and cell lysates were subjected to western immunoblot analysis as described under Materials and methods (**A**). Representative morphology of survivin-sense and antisense-transfected cells is shown in (**B**). For clonogenic assay, cells were seeded in triplicate at a density of 1500 cells per 10 cm dish and allowed to grow for 7–10 days until visible colonies were formed. The results are presented as percentage of the number of colonies formed relative to control (sense survivin treated) dishes (*n*=3). Bars represent mean±s.e.m. Representative dishes of control (survivin sense) and survivin antisense transfected cells are shown in the lower panel. Survivin depletion induced significant inhibition of clonogenic survival, to <90% of control transfected cultures (*P*<0.001).

To examine the role of survivin in RCC cell proliferation and survival we knocked down survivin expression in 786-0 RCC cells using LY2181308 sodium, a specific antisense survivin oligonucleotide. As shown in Figure 5A, transfection of 786-0 cells with LY2181308 sodium resulted in significant reduction in survivin levels as determined by western immunoblotting. Transfection of 786-0 cells with the LY2181308 sodium also resulted in an increase in multinucleated cells (Figure 5A, lower panel). Morphologically, these cells became abnormally large, flattened, and accumulated nuclei. The levels of p21 and cleaved PARP were also elevated. Next, experiments were conducted to assess the effects of survivin knockdown on clonogenic survival measured under anchorage-dependent conditions (Figure 5B). We demonstrated that survivin depletion by ASO induced significant inhibition of clonogenic survival, to ∼5% of control transfected cultures in 786-0 cells (*P*<0.001). The results suggest that survivin is a critical factor for the proliferation, cytokinesis, and survival of RCC cells.

## Discussion

The introduction of molecular targeted therapy has revolutionised treatment of patients with aRCC. However, there has been a lack of data delineating the exact molecular mechanisms accounting for the clinical effects of such therapy in aRCC. In this study, we developed a patient-derived RCC xenograft model to study molecular changes in RCC xenografts in response to treatment by sorafenib. We report the establishment of three patient-derived xenografts from RCC tumours and have shown that these xenografts exhibit cellular and tissue characteristics that are very similar to the original tumours. These tumours include a conventional clear cell RCC, a papillary RCC, and a poorly differentiated clear cell RCC with sarcomatoid changes. A sarcomatoid component can occur in all RCC histological subtypes and signifies an aggressive disease with poor prognosis. Our study shows that the clinical activities of sorafenib are mediated not only through VEGFR and PDGFR, and their principal downstream signalling pathways (Raf/MEK/ERK and PI3K/AKT) but also through inhibition of non-angiogenic pathways that include inhibition of apoptosis, cell cycle proteins, and survivin, which are not previously reported.

In this study, we showed that inhibitions of phosphorylation of VEGFR-2 and PDGFR-*β* were detected in the sorafenib-treated tumours (Figure 4A). It is known that VEGF promotes proliferation, migration, invasion, and survival of endothelial cells (Tran *et al*, 1999) and the migratory process is in part mediated by activation of Raf/MEK/ERK signalling cascades (Graf *et al*, 1997; Pukac *et al*, 1998). It is possible that the potent anti-angiogenic effects of sorafenib in RCC xenograft may therefore be a result of direct functional impairment of tumour-vessel-associated endothelial cells and vascular smooth muscle cells. By disrupting VEGF signalling, sorafenib is able to inhibit VEGF-driven tubular formation, and endothelial cell migration and sprouting, leading to a striking reduction in tumour growth and microvessel density as observed in sorafenib-treated RCC xenografts (Figure 3). The PDGF is angiogenic for microvascular sprouting of endothelial cells, in particular PDGF-BB and receptors recruit pericytes and smooth muscle cells around nascent vessel sprouts (Conway *et al*, 2001). This pericyte–endothelial interaction normally confers resistance to VEGFR antagonists on endothelial cells (Erber *et al*, 2004). Thus, the inhibition of both PDGFR and VEGFR by sorafenib may enhance tumour vessel regression by disruption of the pericyte-mediated endothelial cell survival mechanisms, as shown in Figure 3.

Our findings showed that sorafenib induces comparable tumour inhibition in conventional clear cell (RCC-07-0408), papillary (RCC-02-0908), and poorly differentiated clear cell sarcomatoid (RCC-25-0908) RCCs, suggesting that the clinical effect of sorafenib in RCC is not limited to conventional clear cell RCC subtype. This suggests that inhibition of angiogenic targets (VEGFR and PDGFR) may not be the only mechanism of action that contributes to the clinical effects of sorafenib. Inhibition of non-clear cell RCC by sorafenib could be mediated by inhibition of non-angiogenic pathway, including its effects on cell cycle, apoptosis, and survivin expression.

As for the effects of sorafenib on cell cycle in RCC, we showed that sorafenib-induced growth suppression was associated with inhibition of cyclin D1, cyclin B1, and apoptosis induction. As cyclin D1 is required for G1 cell cycle progression, inhibition of cyclin D1 expression by sorafenib would arrest the cells at G1/S phase. Also our present study shows that sorafenib actively induces apoptosis. However, the mechanism(s) responsible for this effect is not clear at the moment. Bim (also known as BCL2-like 11) is a proapoptotic member of the Bcl-2 family implicated in the regulation of apoptosis associated with thymocyte negative selection and following growth factor withdrawal (Whitfield *et al*, 2001; Bouillet *et al*, 2002; Ley *et al*, 2003). It is possible that upregulation of Bim by sorafenib would allow more Bim to bind to and antagonise anti-apoptotic effect of the Bcl-2 and Bcl-xL, leading to Bax-dependent apoptogen release, caspase activation, and cell death.

Lastly, we showed that in addition to inhibition of angiogenic pathways, sorafenib also suppressed survivin expression. Survivin knockdown by survivin-specific ASO inhibited cell growth and induced cell death in clear cell RCC cells. We hypothesised that inhibition of survivin may contribute to the clinical activities of sorafenib, an observation substantiated by the fact that the degree of survivin suppression correlates well with the degree of tumour growth suppression by these drugs. The findings have important clinical implications as this observation renders survivin a target for therapeutic development of aRCC. The mechanistic pathway leading to suppression of survivin by sorafenib is unclear at the moment. It is postulated that the downregulation of survivin was a result of VEGFR/PDGFR inhibition by sorafenib as survivin expression is regulated by TKR activity (Sato *et al*, 2006). However, the exact mechanism of survivin suppression by sorafenib, which could be a direct effect or mediated through other signalling pathways, is currently being investigated.

A study using a murine xenograft model however has its inherent limitations. First, the uptake rate of xenograft in SCID mice in our experience is about 30–40%. Clinical conclusions based on data derived from a limited number of RCC xenografts should be interpreted with caution. Second, even though we demonstrated that the xenografts we generated retain identical histological features compared with the original specimens, tumour behaviour in a non-orthotropic environment in mice may not resemble the microenvironment of RCC in patients. Thus, it is possible that tumour response in a xenograft model may not be reproducible clinically. Nevertheless, our molecular data generated using RCC-derived xenografts is consistent and reproducible, and it sheds light into the molecular mechanisms accounting for the clinical effects of sorafenib in aRCC.

In summary, we have shown that both clear cell and non-clear cell RCCs responded to treatment with sorafenib. In addition, we have presented novel findings on the effects of sorafenib on non-angiogenic targets that include proteins involve in cell cycle regulation, apoptosis, and survivin, which may contribute to the overall clinical activity of sorafenib in RCC, in addition to inhibition of both the AKT and ERK signalling pathways downstream of VEGFR and PDGFR. These non-angiogenic targets could explain the observation that clinical activity of sorafenib in RCC is not limited to the conventional clear cell histological subtype.

## Supplementary Material

Supplementary Figure 1S

Supplementary Figure 2S

Supplementary Figure 3S
