# Supplementary material for: Inhibition of angiogenic and non-angiogenic targets by sorafenib in renal cell carcinoma (RCC) in a RCC xenograft model
Source: Br J Cancer. 2011 Mar 15;104(6):941–7. doi: 10.1038/bjc.2011.55 (PMC3065286; doi:10.1038/bjc.2011.55)
Supplement: Supplementary Figure 1S [file bjc201155x1.ppt]

## Slide 1
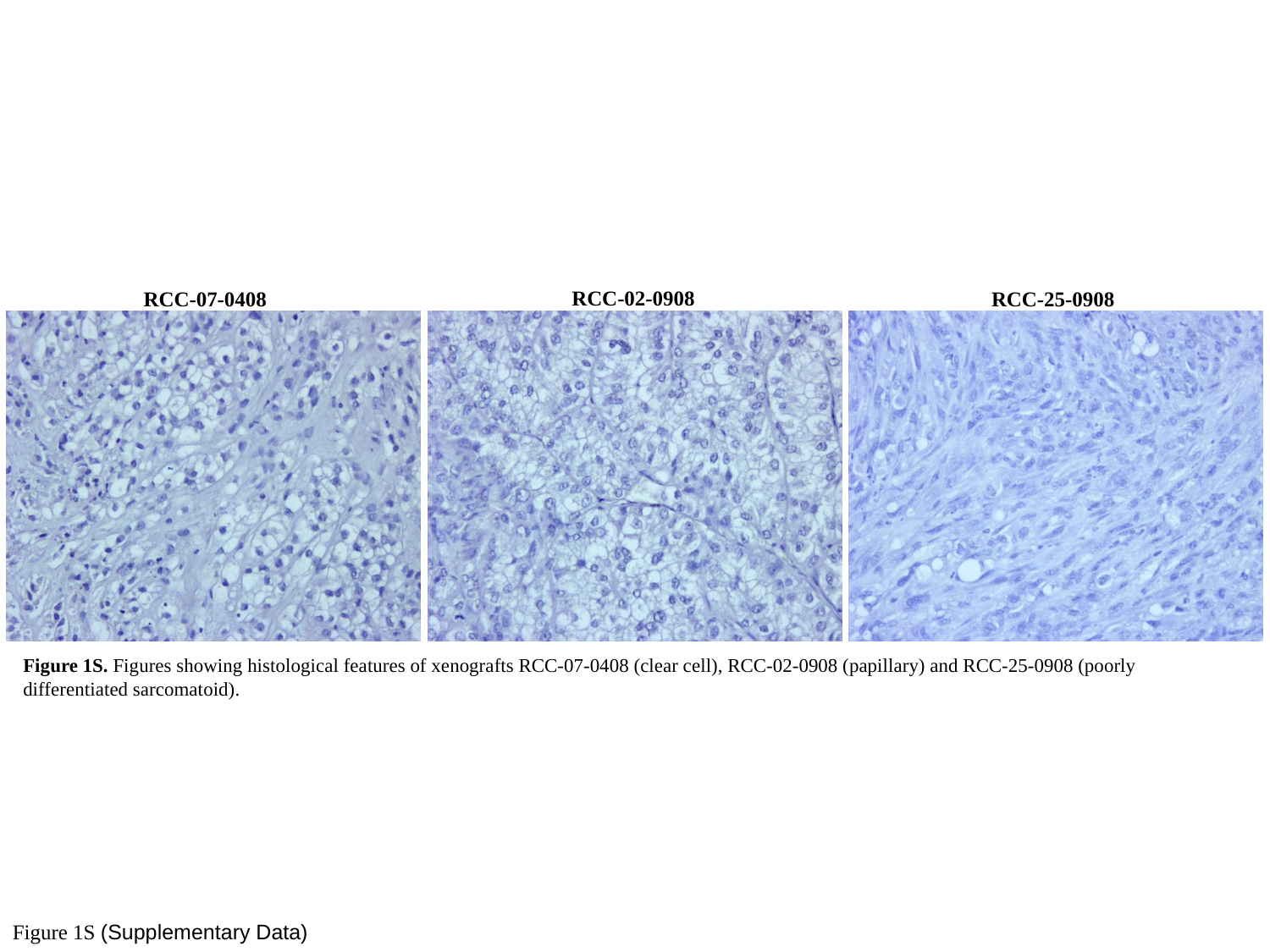

RCC-02-0908
RCC-07-0408
RCC-25-0908
Figure 1S. Figures showing histological features of xenografts RCC-07-0408 (clear cell), RCC-02-0908 (papillary) and RCC-25-0908 (poorly differentiated sarcomatoid).
Figure 1S (Supplementary Data)
