# Supplementary material for: Inhibition of angiogenic and non-angiogenic targets by sorafenib in renal cell carcinoma (RCC) in a RCC xenograft model
Source: Br J Cancer. 2011 Mar 15;104(6):941–7. doi: 10.1038/bjc.2011.55 (PMC3065286; doi:10.1038/bjc.2011.55)
Supplement: Supplementary Figure 2S [file bjc201155x2.ppt]

## Slide 1
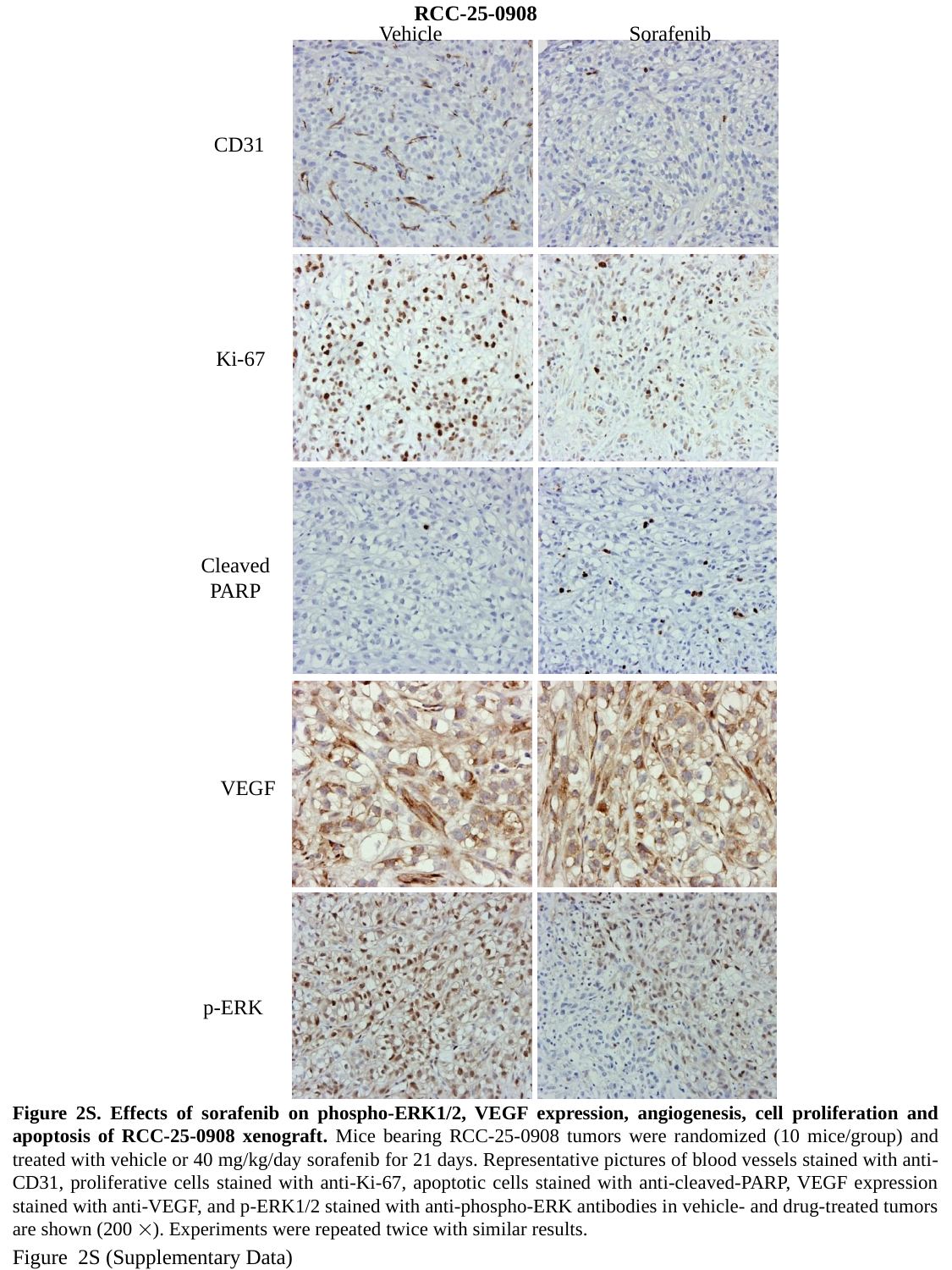

RCC-25-0908
Vehicle
Sorafenib
CD31
Ki-67
Cleaved PARP
VEGF
p-ERK
Figure 2S. Effects of sorafenib on phospho-ERK1/2, VEGF expression, angiogenesis, cell proliferation and apoptosis of RCC-25-0908 xenograft. Mice bearing RCC-25-0908 tumors were randomized (10 mice/group) and treated with vehicle or 40 mg/kg/day sorafenib for 21 days. Representative pictures of blood vessels stained with anti-CD31, proliferative cells stained with anti-Ki-67, apoptotic cells stained with anti-cleaved-PARP, VEGF expression stained with anti-VEGF, and p-ERK1/2 stained with anti-phospho-ERK antibodies in vehicle- and drug-treated tumors are shown (200 ×). Experiments were repeated twice with similar results.
Figure 2S (Supplementary Data)
