# Supplementary material for: Inhibition of angiogenic and non-angiogenic targets by sorafenib in renal cell carcinoma (RCC) in a RCC xenograft model
Source: Br J Cancer. 2011 Mar 15;104(6):941–7. doi: 10.1038/bjc.2011.55 (PMC3065286; doi:10.1038/bjc.2011.55)
Supplement: Supplementary Figure 3S [file bjc201155x3.ppt]

## Slide 1
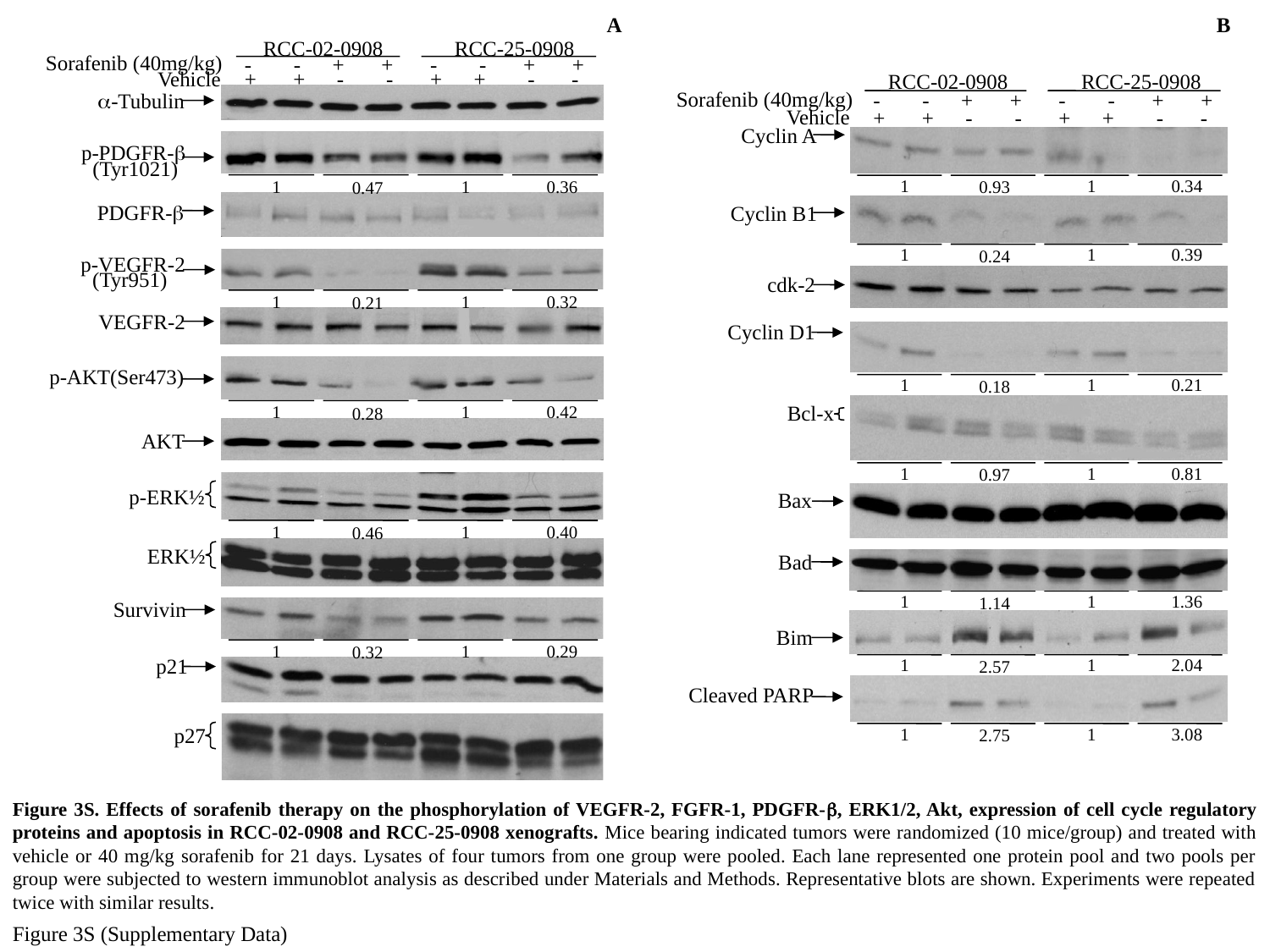

A
B
RCC-02-0908
RCC-25-0908
Sorafenib (40mg/kg)
 - - + + - - + +
 + + - - + + - -
Vehicle
α-Tubulin
p-PDGFR-β
(Tyr1021)
1
1
0.36
0.47
PDGFR-β
p-VEGFR-2
(Tyr951)
1
1
0.32
0.21
 VEGFR-2
p-AKT(Ser473)
1
1
0.42
0.28
AKT
p-ERK½
1
1
0.40
0.46
ERK½
Survivin
1
1
0.29
0.32
p21
p27
RCC-02-0908
RCC-25-0908
Sorafenib (40mg/kg)
 - - + + - - + +
Vehicle
 + + - - + + - -
Cyclin A
1
1
0.34
0.93
Cyclin B1
1
1
0.39
0.24
cdk-2
Cyclin D1
1
1
0.21
0.18
Bcl-x
1
1
0.81
0.97
Bax
Bad
1
1
1.36
1.14
Bim
1
1
2.04
2.57
Cleaved PARP
1
1
3.08
2.75
Figure 3S. Effects of sorafenib therapy on the phosphorylation of VEGFR-2, FGFR-1, PDGFR-β, ERK1/2, Akt, expression of cell cycle regulatory proteins and apoptosis in RCC-02-0908 and RCC-25-0908 xenografts. Mice bearing indicated tumors were randomized (10 mice/group) and treated with vehicle or 40 mg/kg sorafenib for 21 days. Lysates of four tumors from one group were pooled. Each lane represented one protein pool and two pools per group were subjected to western immunoblot analysis as described under Materials and Methods. Representative blots are shown. Experiments were repeated twice with similar results.
Figure 3S (Supplementary Data)
